# Maximising the wrench capability of mobile manipulators with experiments on a UVMS

**DOI:** 10.3389/frobt.2024.1442813

**Published:** 2025-01-30

**Authors:** Wilhelm J. Marais, Oscar Pizarro, Stefan B. Williams

**Affiliations:** ^1^ Australian Centre For Robotics (ACFR), University of Sydney, Sydney, NSW, Australia; ^2^ Department of Marine Technology, Norwegian University of Science and Technology (NTNU), Trondheim, Norway

**Keywords:** underwater manipulation, kinematic redundancy, bi-level optimisation, redundancy parameterisation, wrench maximisation, trajectory optimisation

## Abstract

This paper presents methods for finding optimal configurations and actuator forces/torques to maximise contact wrenches in a desired direction for underwater vehicle manipulator systems (UVMS). The wrench maximisation problem is formulated as a bi-level optimisation problem, with upper-level variables in a low-dimensional parameterised redundancy space, and a linear lower-level problem. We additionally consider the cases of one or more manipulators with multiple contact forces, maximising the wrench capability while tracking a trajectory and generating large wrench impulses using dynamic motions. The specific cases of maximising force to lift a heavy load and maximising torque during a valve-turning operation are considered. Extensive experimental results are presented using a 6 degree of freedom (DOF) underwater robotic platform equipped with a 4DOF manipulator and show significant increases in the wrench capability compared to existing methods for mobile manipulators.

## 1 Introduction

High degree of freedom (DOF) vehicle manipulator systems have seen increased use in both industrial and field robotics settings due to the advantages provided by kinematic redundancy. Since these systems have a large number of DOFs responsible for end effector motion, kinematic positioning generally requires iterative techniques. Despite this, these kinematically redundant systems can make use of the continuous space of configurations, which solve a particular inverse kinematics problem to optimise additional secondary objectives (Klein and Huang, 1983). Secondary objectives may include obstacle avoidance, optimisation of dynamic manipulability, and avoidance of configurations limits (Cieslak et al., 2015; Antonelli, 2005). During interaction tasks with objects or the environment, end effector poses and contact wrenches are of importance (Khatib, 1987; Antonelli et al., 2001). External contact forces and torques between the end effector and the environment are transmitted through each DOF of the system responsible for end effector motion, in a way which is highly dependent on the configuration. Exploiting the continuous redundancy offered by high-DOF systems allows for configurations which simultaneously achieve a desired end effector pose and maximise the wrench capabilities (Busson and Béarée, 2018).

Interaction tasks in underwater environments for scientific exploration or industrial purposes have been traditionally conducted by medium-to-large underwater vehicle manipulator systems (UVMS), requiring specialised equipment and multiple operators for launch and recovery, as well as operation (Sivčev et al., 2018). In recent years, the emergence of smaller and lower cost commercial off-the-shelf underwater vehicles and manipulators has seen a transition to small vehicles for some interaction tasks (Bezanson et al., 2017; Carlucho et al., 2021). This reduces both operation costs and time and increases the accessibility of these systems since they can be launched, operated, and recovered by a small team without specialised equipment. This work improves the capability of these small systems, allowing them to perform a larger range of interaction tasks. Specifically, we consider maximising contact wrenches between the end effector and the environment. This work assumes the object to be manipulated has already been firmly grasped by the end effector of the UVMS, and the system can then transition to a configuration which allows for the maximum wrench to be applied at the end effector.

We compare a number of cases whose wrench capabilities should be maximised. The first is the static case, where the aim is to maximise the wrench at a single configuration, which we further extended to consider multiple contact points with the environment. The second case considers a trajectory where a given desired end effector path should be tracked while finding a set of configurations along the way which are dynamically feasible and maximise the lowest applicable wrench along the path. Finally, the case of generating large wrench impulses for a fixed end effector pose is considered. Extensive experimental validation of the proposed methods is provided, which shows significant increases in the wrench capability compared to previous methods for UVMS and other mobile manipulators.

This paper presents the following contributions:

• A bi-level optimisation approach for finding optimal configurations and actuator efforts for maximising wrenches for UVMS. Experimental results show this provides significantly larger wrenches than existing transmission ratio optimisation methods.

• Consideration of relaxing constraints on orthogonal wrenches for certain tasks, with experimental results showing a threefold increase in the maximum wrench capability.

• An optimisation method for maximising wrenches for UVMS with multiple contact points, including parameterisation of a set of secondary grasping points and analysis of the required constraints. Experimental results show increased wrench capability using the proposed methods.

• A bi-level optimisation method for generating whole-body trajectories which maximise wrenches over a pre-defined end effector trajectory, with experimental results confirming the validity of the proposed method.

• A proposed method for generating dynamic motions with a fixed end effector pose for generating large wrench impulses, with experimental results showing the validity of the approach.

The rest of this paper is structured as follows: Section 2 provides a recap of the mathematical background and current methods for wrench analysis and finding optimal configurations for wrench maximisation. Section 3 describes the methods proposed in this work for maximising the static wrench capability for UVMS, including consideration of multiple contact points. Section 4 extends the methods used for static analysis to consider dynamic trajectories, given an end effector path. Section 5 describes the proposed method for generating large wrench impulses using dynamic motions. Finally, Section 6 presents experimental results for each section, followed by concluding remarks and directions for future work in Section 7.

## 2 Background and related work

### 2.1 Kinematic and dynamic modelling

**FIGURE 1:**
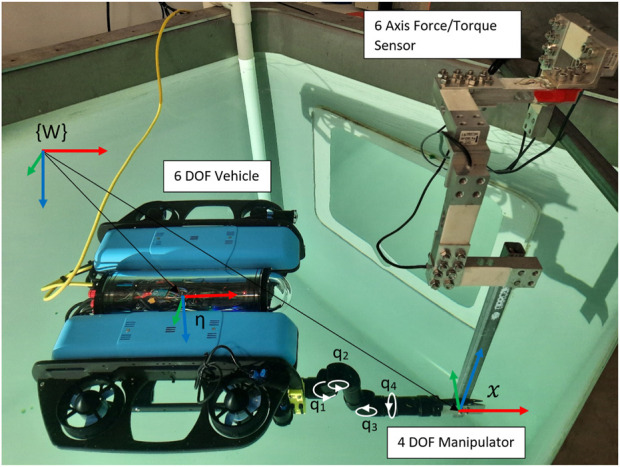
UVMS used in experiments with a 6DOF BlueROV vehicle and 4DOF Reach Robotics Alpha manipulator, showing vehicle pose η and end effector pose x relative to the world frame {W} . Manipulator joints are labelled $q_{1}$ to $q_{4}$ .

A vehicle manipulator system has system configuration $\theta ={\left(\eta ,q\right)}^{T}$ , with dim(θ)=n , where η∈SE(3) is the vehicle pose in the world frame and $q\in R^{n-6}$ represents the manipulator joint angles. These are shown in Figure 1. Given some end effector pose x∈SE(3) , in the world frame, the forward non-linear map $f_{k}$ gives Equation 1

$$x=f_{k}\left(\theta \right),$$ (1)

for which analytical solutions in the reverse direction, the inverse kinematics problem, are generally not available. Numerical solutions make use of the linear velocity relationship Equation 2

$$\dot{x}=J\left(\theta \right)\dot{\theta },$$ (2)

where $\dot{x}\in R^{6}$ is the end effector velocity vector in the inertial frame, $\dot{\theta }\in R^{n}$ is the vector of system velocities corresponding to each DOF, and J(θ) is the configuration-dependent Jacobian. The θ dependence for J is dropped in further notation. For kinematically redundant systems, this has a least squares solution for a desired $\dot{x}$ given by Equation 3

$$\dot{\theta }=J^{†}\dot{x},$$ (3)

where $J^{†}$ is the pseudoinverse of J . Note in this work, we consider fully actuated vehicles; therefore, the Jacobian for a single manipulator system always has a full row rank. The null space of J , defined as the set of system velocities which cause no end effector velocity, can be included as

$$\dot{\theta }=J^{†}\dot{x}+\alpha \left(I-J^{†}J\right)\nabla h,$$ (4)

where α is a scaling factor and h represents some secondary objective to be optimised in the null space (Klein and Huang, 1983). The null space projection $\left(I-J^{†}J\right)$ can be assumed as a set of (possibly non-linearly independent) basis vectors, which is tangent to the inverse kinematics equality constraint.

Now, we have the end effector wrench vector in Equation 5

he=fe0ne0∈R6, (5)

consisting of end effector forces fe0 and torques ne0 in the inertial frame, with the force relationship

$$\tau_{h}=J^{T}h_{e},$$ (6)

where $\tau_{h}\in R^{n}$ represents the vector of forces and torques on each DOF due to $h_{e}$ . The actuator model is given by Bu (Antonelli, 2005), where $u\in \left[u_{\min},u_{\max}\right]\in R^{m}$ is the control input vector for each actuator, and the matrix $B\in R^{n\times m}$ is the mapping between the actuator control input and the resultant force/torque on each DOF of the system. Including the dynamics of the system gives (Antonelli, 2005)

$$M\left(\theta \right)\ddot{\theta }+C\left(\theta ,\dot{\theta }\right)\dot{\theta }+D\left(\theta ,\dot{\theta }\right)\dot{\theta }+g\left(\theta \right)+J^{T}h_{e}=Bu,$$ (7)

where M(θ) is the configuration-dependent mass matrix, $C\left(\theta ,\dot{\theta }\right)$ is the Coriolis term, $D\left(\theta ,\dot{\theta }\right)$ is the damping term modelled using a combination of linear and quadratic drags, and g(θ) is the vector of gravity and buoyancy forces. We compile all dynamic terms on the left into the vector $\tau_{d}$ , yielding

$$\tau_{d}+J^{T}h_{e}=Bu.$$ (8)

### 2.2 Wrench ellipsoids

Analysis of wrench capability typically involves ellipsoids and polytopes. Velocity ellipsoids and their counterparts, wrench ellipsoids, were introduced by Yoshikawa (1985). These provide a mapping from a unit ball of joint torques to the end effector wrench, according to Equation 6. Care must be taken to avoid physical inconsistency due to the use of norms and inner products on wrench and axis force/torque spaces (Duffy, 1990). The use of appropriate scaling metrics (Doty et al., 1993; Doty et al., 1995) gives a modified version of Equation 6 expressed as Equation 9

$$W_{\tau }^{1/2}\tau_{h}=\left(W_{\tau }^{1/2}J^{T}W_{h}^{-1/2}\right)W_{h}^{1/2}h_{e},$$ (9)

where $W_{\tau }$ and $W_{h}$ are the scaling metrics for $\tau_{h}$ and $h_{e}$ , respectively (Doty et al., 1993). The result is that the vector spaces $W_{\tau }^{1/2}\tau_{h}$ and $W_{h}^{1/2}h_{e}$ have physically consistent inner products and norms. In this work, we arbitrarily consider identity scaling metrics, i.e., $W_{\tau }=I_{n}$ and $W_{h}=I_{6}$ , where $I_{n}$ and $I_{6}$ are the n×n and 6×6 identity matrices, respectively, where the elements of each matrix have the appropriate units to provide physical consistency (see Doty et al. (1993) for an in-depth discussion).

Dropping the scaling metrics for brevity, the wrench ellipsoid is defined as Equation 10

$$\left\{h_{e}\;|\;\|J^{T}h_{e}{\|}_{2}\leq 1\right\}.$$ (10)

Recent work proposed maximising the volume of the velocity ellipsoid by projecting the gradient onto the null space of the system, as shown in Equation 4, and by further solving the problem as a quadratic program (Zhang et al., 2016). A similar null space projection method was used by Bae et al. (2018) to maximise the dynamic manipulability ellipsoid during a value-turning operation for a UVMS.

### 2.3 Transmission ratios

The volume of the wrench ellipsoid accounts for the ability of the system to apply forces and torques in all directions in an isotropic manner. In this work, it is desired to maximise the wrench along a single force/torque direction; therefore, anisotropic capability measures are more appropriate. One such measure is the transmission ratio—which is defined as the distance to the wrench ellipsoid from the origin in a desired direction—which can been optimised by null space projection (Faroni et al., 2016).

To determine the distance to the wrench ellipsoid in a given direction, consider a unit vector $\hat{c}$ , which defines the desired wrench, according to the metric $W_{h}^{-1/2}\hat{c}$ , as defined above. Again, in this work, we consider identity scaling metrics, so $W_{h}$ and $W_{\tau }$ are dropped in further notation, yet they are required to avoid physical inconsistency and allow the use of norms and unit vectors. Now, considering a scalar β , which scales the wrench in the direction of $\hat{c}$ , with the condition that the result lies on the ellipse given by Faroni et al. (2016) in Equation 11,

$${\left(\beta \hat{c}\right)}^{T}JJ^{T}\left(\beta \hat{c}\right)=1$$ (11)

and solving for β in Equation 12,

$$\beta ={\left({\hat{c}}^{T}JJ^{T}\hat{c}\right)}^{-1/2}.$$ (12)

This analysis does not include the effect of gravity, buoyancy, and dynamical effects, which offsets the centre of the ellipsoid, yielding a dynamic wrench ellipsoid defined as dynamic wrench ellipsoid defined in Equation 13

$$\left\{h_{e}\;|\;\|J^{T}h_{e}+\tau_{d}{\|}_{2}\leq 1\right\}.$$ (13)

For an ellipse with its centre not at the origin, we can solve for β , assuming the origin is contained in the ellipsoid. This condition is met with a system which can support its own weight under gravity and sustain the dynamic loads. Solving Equation 14

$${\left(J^{T}\beta \hat{c}+\tau_{d}\right)}^{T}\left(J^{T}\beta \hat{c}+\tau_{d}\right)=1,$$ (14)

for β simply involves taking the positive root. This definition does not account for the actuator mapping B or actuator limits. Symmetric torque limits have been proposed to be incorporated into the transmission ratio (Xing et al., 2020), yet dynamical effects were neglected. Some works have considered minimising the sum of squared torques on each joint for a given wrench of a redundant serial manipulator by null space projection (Nemec, 1997), which is functionally equivalent to optimising the transmission ratio.

The idea of wrench ellipsoids was extended by Ajoudani et al. (2017) by scaling the ellipsoid by a desired force/stiffness matrix and instead optimising the trace of the resultant ellipsoid (the sum of the square of the radii) to obtain a configuration-dependent measure. Bowling and Khatib (2005) explored combining velocity, acceleration, and wrench capabilities in the joint torque space for serial manipulators. This involves mapping maximal balls of velocity, acceleration, and wrench individually onto ellipsoids in the joint torque space and then combining to form a torque hypersurface, which has to satisfy actuator constraints. Since the method combines worst-case scenarios for velocities, accelerations, and wrenches, it provides a very conservative measure of the manipulator capability.

### 2.4 Wrench polytopes

It has been shown that wrench ellipsoids provide only an approximate measure of manipulator performance as it fails to capture the true constraints of the system, as compared to wrench polytopes, which provides a better description of the true capabilities of a system (Chiacchio et al., 1996). By finding the set of effector wrenches which satisfy joint constraints, the wrench polytope is define in Equation 15

$$\left\{h_{e}\;|\;\tau_{\min}\leq \left(J^{T}h_{e}+\tau_{d}\right)\leq \tau_{\max}\right\},$$ (15)

where $\tau_{\min}$ and $\tau_{\max}$ are the minimum and maximum loads on each DOF, respectively. Wrench polytope analysis has been used extensively in the design and evaluation of manipulators and manipulator poses (Firmani et al., 2008; Boudreau et al., 2021), yet optimisation is difficult since the quality of a polytope is difficult to quantify. Some attempts to define capability measures using polytopes involve computing actuator saturation for a given wrench direction for serial manipulators (Busson and Béarée, 2018), or solving linear programming (LP) problems for parallel manipulators (Buttolo and Hannaford, 1995; Lin and Chen, 2016), although these methods are not directly applicable to mobile manipulator systems.

### 2.5 Redundancy parameterisation

The idea of redundancy parameterisation has been proposed in several works to greatly reduce the number of dimensions over which a given performance objective needs to be optimised, as well as to remove the non-linear inverse kinematics constraint. These methods avoid the use of the Jacobian null space projection in Equation 4 and instead explicitly consider a minimal set of DOF, which describe the available self-motions while keeping a fixed end effector pose. Redundancy parameterisation has been used for choosing optimal stiffness configurations in serial manipulators with one degree of redundancy (Busson et al., 2017), as well as for exploring the force capabilities of a manipulator to apply forces normal to a surface (Nützi et al., 2013). This work was extended in Busson and Béarée (2018) to an exhaustive search over two redundant DOFs for a 7DOF manipulator, where the saturating wrench was used to find the weightlifting capability for each pose, while Boudreau and Nokleby (2012) examined a planar parallel manipulator with a 3DOF redundant space to minimise the sum of squared joint torques for a given wrench. Given an appropriate parameterisation of the redundant DOF written as $\theta_{r}\in R^{n-6}$ , Equation 4 can be rewritten as

$$\dot{\theta }=J^{†}\dot{x}+Z_{r}\dot{\theta_{r}},$$ (16)

where $\dot{\theta_{r}}\in R^{n-6}$ is the vector of velocities of the redundant DOF and $Z_{r}\in R^{n\times \left(n-6\right)}$ is the null space projection matrix, which maps redundant velocities to system velocities, according to the parameterisation.

**FIGURE 2:**
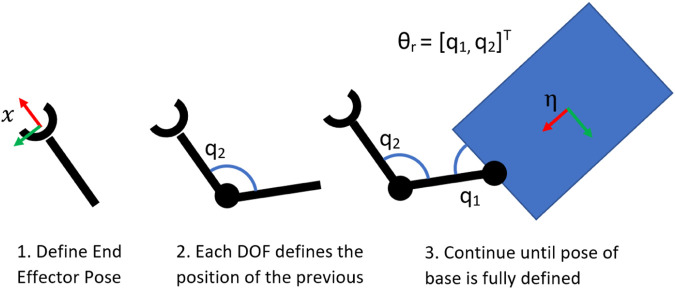
Sequence of images showing the proposed redundancy parameterisation method for UVMS, with redundancy parameters $\theta_{r}={\left[q_{1},q_{2}\right]}^{T}$ .

A redundancy parameterisation method for UVMS was introduced by Marais et al. (2021), which is illustrated in Figure 2. Given a desired end effector pose, each DOF in reverse order starting from the end effector is a redundant DOF until the pose of the base is fully defined, with invalid poses due to self-collision discarded. The redundant configuration $\theta_{r}$ , together with the end effector pose x , can be used to determine the full system configuration θ .

### 2.6 Wrench capability over a trajectory

The previous analysis has considered wrench capabilities for a single end effector pose. In some cases, the wrench capability over an entire trajectory has to be considered. Local redundancy resolution methods have been used for continuous wrench maximisation over a trajectory (Bae et al., 2018), yet local methods may lead to numerical instability and sub-optimal trajectories (Kazerounian and Wang, 1988). Global redundancy resolution methods which consider entire paths have been proposed for trajectory planning in kinematically redundant systems (Suh and Hollerbach, 1987). Early works in global redundancy resolution (Papadopoulos and Gonthier, 1995a; Papadopoulos and Gonthier, 1995b) focused on planning paths, which avoid actuator saturation while experiencing a large fixed load. Further work used the intersection of the wrench polytope with an expected cone of disturbances for generating trajectories for kinematic manipulators which are robust to disturbances (Ferrolho et al., 2021; Lin and Chen, 2016) and used an evolutionary algorithm combined with multiple lower-level linear programming solutions to find the maximum wrench capability over a trajectory for a re-configurable parallel robot. An iterative linear programming method was proposed for trajectory optimisation to maximise the allowable load for mobile manipulators between two end effector set points (Korayem et al., 2004), yet the computational complexity restricted its application to a 2DOF manipulator.

Hamiltonian control approaches, which make use of Pontryagin’s minimum principle over an entire trajectory (Korayem et al., 2009; Korayem et al., 2012), have been used for finding the maximum load carrying capacity for mobile manipulators yet often result in control laws which require instantaneous switching of actuator forces or torques, which are not physically realisable for the systems considered in this work. Additionally, these methods require solving two-point boundary value problems and, therefore, are generally restricted to systems with low degrees of redundancy. Recent work has considered receding horizon style planning using model predictive control (MPC) to track desired wrenches (Wahrburg and Listmann, 2016), or for trajectory planning for a mobile manipulator actuating a large load (Sleiman et al., 2021). Other works Li and Nguyen (2023) have considered kinodynamic pose optimisation for whole body control of a humanoid robot manipulating an object, with real-time trajectory tracking formulated as a constrained MPC problem, yet few of these works have considered explicitly maximising the wrench capability using these methods.

To the best of the authors' knowledge, no effective methods exist for explicitly maximising the wrench capability of high DOF vehicle manipulator systems, which appropriately consider and exploit the full capabilities and constraints which are unique to these systems.

## 3 Maximising the static wrench capability

This section details how to find optimal configurations and actuator efforts to maximise the wrench capability in a desired direction, for a single end effector pose and system configuration. Dynamic effects due to velocity and acceleration are, therefore, ignored yet are considered in Section 4. Cases in which the maximising static wrench capabilities are relevant include lifting a heavy load or maximising instantaneous torque when turning a valve.

### 3.1 Problem formulation

The static wrench maximisation problem can be expressed as Equation 17

$$\underset{\theta ,u,h_{e}}{\max}\;\;{\hat{c}}^{T}W_{h}^{1/2}h_{e},$$ (17)

with constraints

$$x_{d}=f_{k}\left(\theta \right),$$ (18)

$$\theta_{\min}\leq \theta \leq \theta_{\max},$$ (19)

$$d_{\min}\left(\theta \right)>0,$$ (20)

$$u_{\min}\leq u\leq u_{\max},$$ (21)

$$\tau_{d}+J^{T}h_{e}=Bu,$$ (22)

$$\left(\hat{c}{\hat{c}}^{†}-I_{6}\right)W_{h}^{1/2}h_{e}=0,$$ (23)

where as before, θ , u , $h_{e}$ , $\tau_{d}$, and J are the system configuration, actuator effort, end effector wrench, vector of dynamic terms, and system Jacobian, respectively, described in Section 2. Both $\tau_{d}$ and J have a dependence on θ . The desired end effector pose is given by $x_{d}\in SE\left(3\right)$ ; the terms $u_{\min}$ and $u_{\max}\in R^{m}$ are minimum and maximum actuator efforts, respectively; $W_{h}$ is the wrench scaling metric as before, and $\hat{c}\in R^{6}$ is the unit vector, as shown in Section 2, which describes the direction of force/torque, in which the wrench should be maximised. The first constraint in Equation 18 is the inverse kinematics constraint using the forward non-linear map $f_{k}$ , Equation 19 considers configuration limits, and the constraint in Equation 20 is to prevent self-collisions and collisions with the environment using the stand-in function $d_{\min}\left(\theta \right)$ , which is simply the minimum distance from self-collision or between each link of the UVMS system and obstacles in the environment. Equations 21, 22 are the actuator limits and static balance constraints, respectively. Finally, Equation 23, where $I_{6}$ is a 6×6 identity matrix, enforces $W_{h}^{1/2}h_{e}$ to have no force or torque components orthogonal to $\hat{c}$ . As in Section 2, care has to be taken when considering orthogonality of wrench vectors (Duffy, 1990), hence the use of the scaling metric.

This formulation has a linear objective with combined linear and non-linear constraints and is intractable to solve in a reasonable timescale using available non-linear solvers for the system considered in this work. In particular, satisfying the inverse kinematics equality constraint in Equation 18 is difficult due to the absence of analytical solutions for kinematically redundant systems. This work proposes two methods to make solving the above problem tractable: redundancy parameterisation and separation into a bi-level optimisation problem. Bi-level optimisation has been used in several works on kinematically redundant manipulators to make optimisation problems tractable (Nusbaum et al., 2020; Menasri et al., 2013; Menasri et al., 2015). Typically, the problem is partitioned between two sets of decision variables and solved in a nested framework (Dempe, 2002). This work uses the proposed redundancy parameterisation method in Marais et al. (2021), allowing the inverse kinematics constraint in Equation 18 to be explicitly satisfied and, therefore, removed from the list of constraints and replacing θ with $\theta_{r}$ , the terms in redundancy parameterisation. Next, we recognise the following: the dynamic balance constraint in Equation 22 is linear in u and $h_{e}$ for a given θ . Therefore, Equations 21–23 are all linear in u and $h_{e}$ . Coupled with the objective which is similarly linear, the problem naturally separates into a linear lower-level problem with decision variables u and $h_{e}$ , and a non-linear upper-level problem with the decision variable $\theta_{r}$ . Rewriting the newly formulated bi-level optimisation problem as an upper-level problem Equation 24

$$\underset{\theta_{r}}{\max}\;\beta_{2}^{*},$$ (24)

with constraints in Equations 25, 26

$$\theta_{r_{\min}}\leq \theta_{r}\leq \theta_{r_{\max}},$$ (25)

$$d_{\min}\left(\theta_{r}\right)>0,$$ (26)

where $\beta_{2}^{*}$ is the solution to the lower-level problem

$$\beta_{2}^{*}=\underset{u,h_{e}}{\max}\;{\hat{c}}^{T}W_{h}^{1/2}h_{e},$$ (27)

with constraints given in Equations 21–23.

It must be noted that for certain sets of upper-level variables, the lower-level problem may not have a feasible solution. Physically, this corresponds to configurations where the system is unable to hold a static configuration due to external forces such as gravity and buoyancy acting on the system. Although not an issue for the system considered in this work, programmatically, these configurations are penalised with a large negative cost. This is further discussed for the case of dynamic trajectories in Section 4.

### 3.2 Analysis using ellipsoids and polytopes

**FIGURE 3:**
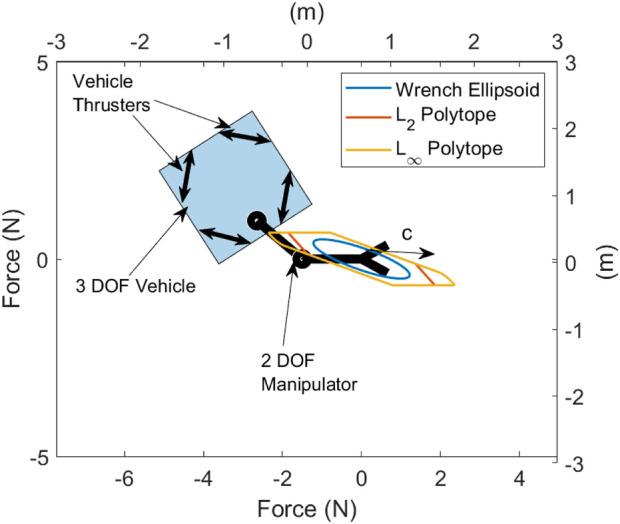
Wrench ellipsoid and polytopes measured in Newton (N) for a 2D UVMS with dimensions in metres (m), showing the force capability in the given configuration and the desired direction c , in which the wrench capability should be maximised.

First, the previous definitions of wrench ellipsoids and polytopes have to be extended to include the overactuated mapping given in Equation 7. We can invert the mapping in Equation 8 to obtain Equation 28

$$u=B^{†}\left(\tau_{d}+J^{T}h_{e}\right),$$ (28)

giving a new definition for the wrench ellipsoid Equation 29

$$\left\{h_{e}\;|\;{\left\|T_{u}B^{†}\left(J^{T}h_{e}+\tau_{d}\right)\right\|}_{2}\leq 1\right\},$$ (29)

where the actuator weighting matrix (Xing et al., 2020) is given by $T_{u}=diag\left(1/min\left\{\left|u_{\min}|,|u_{\max}\right|\right\}\right.$ . The transmission ratio is also redefined as the positive root of Equation 30

$${\left(T_{u}B^{†}\left(J^{T}\beta \hat{c}+\tau_{d}\right)\right)}^{T}\left(T_{u}B^{†}\left(J^{T}\beta \hat{c}+\tau_{d}\right)\right)=1,$$ (30)

for a given desired wrench along the direction $\hat{c}$ . Using the inverted actuator mapping to redefine the wrench polytope gives Equation 31

$$\left\{h_{e}\;|\;u_{\min}\leq B^{†}\left(J^{T}h_{e}+\tau_{d}\right)\leq u_{\max}\right\},$$ (31)

although $B^{†}$ , the pseudoinverse of B , does not give a true measure of the capabilities of the system. Thus, this is referred to as the $L_{2}$ polytope, while the $L_{\infty }$ polytope is given by Equation 32

$$\left\{h_{e}\;|\;Bu=J^{T}h_{e}+\tau_{d},\;u_{\min}\leq u\leq u_{\max}\right\},$$ (32)

which gives a true measure of the actuator capabilities. A simple 2-dimensional (2D) test case is considered to compare each capability measures shown in Figure 3. This shows an underwater vehicle with diagonally mounted thrusters on each corner which can apply 1 N thrust. The arm has two joints, of which each can apply a torque of 1 Nm. The wrench ellipsoid $L_{2}$ and $L_{\infty }$ polytopes along the 2D force dimensions with 0 end effector torque are shown. The $L_{\infty }$ polytope extends as far or further than the $L_{2}$ polytope in all directions and, therefore, gives a better measure of the true wrench capabilities of the system.

### 3.3 Optimising for the maximum wrench capability

Now, consider finding the configuration and combination of thruster forces and joint torques which maximises the wrench capability of the system shown in Figure 3 in the direction $\hat{c}$ while keeping the same end effector pose.

**FIGURE 4:**
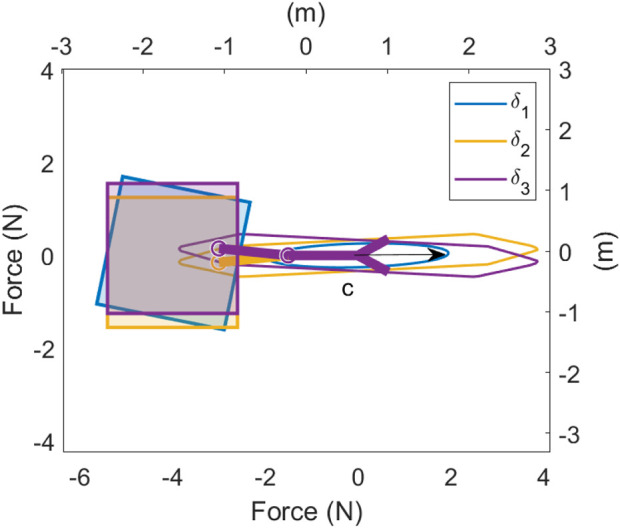
Optimal configurations for a wrench in the c direction for a 2D UVMS, resulting in different optimal configurations according to the measure of wrench capability which is optimised.

For the system considered in Figure 3, there are three vehicle DOFs, two manipulator DOFs, four vehicle thrusters, two joint torques, and three wrench DOFs for a total of 14 variables to solve while simultaneously needing to satisfy the inverse kinematics constraint. Now, consider solving the optimisation problem using the bi-level approach proposed above, separating the problem into a linear programming problem with nine variables (four vehicle thrusters, two joint torques, and three wrench DOFs), and an upper-level problem with two variables (two manipulator DOFs). In this case, redundancy parameterisation has resulted in a bi-level optimisation problem with three fewer total variables to solve. Due to the low dimensionality of the upper-level problem, a simple grid search is used to search the configuration space for the globally optimal configuration. The lower linear programming problem is solved using the dual-simplex method in MATLAB’s linprog function. The optimal solution is shown in Figure 4 labelled $\beta_{2}$ , with the corresponding $L_{\infty }$ polytope. The linear program in Equation 27 can be assumed as finding the value of the $L_{\infty }$ polytope in the direction of $\hat{c}$ . Therefore, the proposed bi-level programming approach can be considered an optimisation over the redundant DOF to optimally configure the system to maximise the $L_{\infty }$ polytope in the direction of $\hat{c}$ .

### 3.4 Removing the orthogonal wrench constraint

The orthogonal wrench equality constraint in Equation 23 can be removed in some scenarios. One example is during a valve-turning operation, where reaction forces orthogonal to $\hat{c}$ , the desired wrench maximisation direction, can be applied on the valve. This enables larger wrenches to be applied in the direction of $\hat{c}$ and results in the same bi-level optimisation problem as before, except with the constraint in Equation 23 removed. The solution to this optimisation problem is shown in Figure 4 labelled $\beta_{3}$ , with the corresponding $L_{\infty }$ polytope. In this case, the bi-level programming approach can be considered an optimisation over the redundant DOF to optimally configure the system such that the $L_{\infty }$ polytope has a maximum extent in the direction of $\hat{c}$ .

### 3.5 Comparison with the transmission ratio

Typical methods for maximising the wrench capability seek to maximise the transmission ratio (Faroni et al., 2016; Nemec, 1997). Considering that the proposed bi-level programming approach determines how to optimally configure the $L_{\infty }$ wrench polytope, consider instead finding the configuration which maximises the transmission ratio. The same bi-level programming approach can be used as before, with a modified lower-level problem which instead uses the transmission ratio. This is given by the following optimisation problem in Equation 33:

$$\underset{\theta_{r}}{\max}\;\beta_{1}^{*},$$ (33)

with constraints in Equations 34, 35

$$\theta_{r_{\min}}\leq \theta_{r}\leq \theta_{r_{\max}},$$ (34)

$$d_{\min}\left(\theta_{r}\right)>0,$$ (35)

where $\beta_{1}^{*}$ is the solution to the lower-level problem in Equation 36

$$\beta_{1}^{*}=\left\{max\;\beta_{1}\;|\;\|T_{u}B^{†}\left(J^{T}\beta_{1}\hat{c}+\tau_{d}\right){\|}_{2}\leq 1\right\}.$$ (36)

In this case, $\beta_{1}^{*}$ is the maximum wrench in the direction of $\hat{c}$ using the transmission ratio and is found by solving for the positive root of the above quadratic equation.

Figure 4 shows the results of bi-level optimisation using each of these objectives $\beta_{1}$ (maximising the transmission ratio), $\beta_{2}$ (maximising the $L_{\infty }$ wrench polytope purely in the direction of $\hat{c}$ ), and $\beta_{3}$ (maximising the $L_{\infty }$ wrench polytope with orthogonal wrench constraints removed) for the same system as in Figure 3 via a grid search over the two redundant DOFs, as well as the corresponding ellipsoid or polytopes. Surprisingly, the optimal configuration changes depending on the lower-level objective used in the bi-level optimisation formulation, despite only two degrees of redundancy. There is also a significant difference in the wrench capability. Maximising $\beta_{1}$ gives a maximum force of 1.91N , while $\beta_{2}$ gives 3.47N and $\beta_{3}$ gives 3.88N . These differences become more pronounced with higher DOF systems, such as the UVMS used for experiments in Section 6. For higher dimensional redundant spaces such as the UVMS considered in this work, simulated annealing followed by a local interior-point method is used to search for an approximately globally optimal solution to the upper-level problem of bi-level optimisation. This is because the upper-level objective is non-smooth and highly non-linear with multiple local maxima, preventing the effective use of gradient-based methods yet with a relatively small number of decision variables (4 for the system in this work). This problem is solvable in several seconds on standard laptop hardware, making it amenable to real-time implementation (assuming the desired end effector pose and wrench maximization direction are defined).

### 3.6 Multiple contact points

The case of multiple contact points is common when using two or more manipulators. The reaction forces of the end effector of a second manipulator on some nearby grasp point can help increase the wrench applied by the first manipulator. This creates a closed kinematic chain between the UVMS system and the environment, requiring more careful analysis.

For multiple contact points’ case, we define $h_{e_{2}}\in R^{6}$ , which is the end effector wrench of the secondary arm, and $C_{2}\in R^{6\times l}$ , which is a set of l≤6 unit vectors, which define the direction in which the second manipulator can apply a wrench $h_{e_{2}}$ . This is explained in more detail below. The force/torque balance equation is given by Equation 37

$$\tau_{d}+J^{T}{\left(h_{e},h_{e_{2}}\right)}^{T}=Bu,$$ (37)

where the terms $\tau_{d},J,B$ and u reflect the additional actuators and DOF of the second manipulator. In order for ${\left(h_{e},h_{e_{2}}\right)}^{T}$ to be well-defined, $J\in R^{12\times n}$ must have a full row rank. This condition is due to the requirement that the system must be able to apply a virtual displacement for a given wrench to be achievable and is violated at kinematically singular configurations. During searches for optimal configurations, the manipulability measure (Yoshikawa, 1985) is used to discard singular or very near singular configurations.

The redundancy parameterisation for the dual manipulator case is similar to the single-arm case, with the primary arm again defining all degrees of redundancy until the pose of the base is fully defined. The inverse kinematics solution to reach the grasp point for the second arm can then be analytically solved. In the case of multiple inverse kinematics solutions for the second arm, the solution which maximises the lower-level objective is taken. In case of no solution, the configuration is considered invalid. The resultant bi-level optimisation problem is written as Equation 38

$$\underset{\theta_{r}}{\max}\;\beta_{2}^{*}$$ (38)

with constraints in Equations 39–42

$$\theta_{r_{\min}}\leq \theta_{r}\leq \theta_{r_{\max}},$$ (39)

$$d_{\min}\left(\theta_{r}\right)>0,$$ (40)

$$\mu \left(\theta_{r}\right)>\mu_{\min},$$ (41)

$$f_{k}^{-1}\left(x_{d},\theta_{r}\right)\neq \emptyset ,$$ (42)

where $\beta_{2}^{*}$ is the solution to the lower-level problem in Equation 43

$$\beta_{2}^{*}=\underset{u,h_{e},h_{e_{2}}}{\max}\;{\hat{c}}^{T}W_{h}^{1/2}h_{e},$$ (43)

subject to constraints in Equations 44, 45

$$u_{\min}\leq u\leq u_{\max},\;\tau_{d}+J^{T}{\left(h_{e},h_{e_{2}}\right)}^{T}=Bu,$$ (44)

$$\left(\hat{c}{\hat{c}}^{†}-I\right)W_{h}^{1/2}h_{e}=0,\;\left(C_{2}C_{2}^{†}-I\right)W_{h}^{1/2}h_{e_{2}}=0.$$ (45)

Again, the decision variables for the upper-level problem are the redundancy parameters $\theta_{r}$ . The term $\mu \left(\theta_{r}\right)$ is the configuration-dependent manipulability measure (Yoshikawa, 1985), which must be greater than some empirically chosen minimum $\mu_{\min}$ to ensure a kinematically deterministic wrench balance configuration. The final upper-level constraint requires the existence of a solution to the inverse kinematics problem $f_{k}^{-1}\left(x_{d},\theta_{r}\right)$ for desired end effector poses $x_{d}\in R^{12}$ . As before, there is an equality constraint which ensures $h_{e}$ has no component orthogonal to c . The final equality constraint ensures $W_{h}^{1/2}h_{e_{2}}$ only has components in allowed directions defined in $C_{2}$ . For example, when firmly grasping a contact point with the secondary manipulator, it might be feasible to set $C_{2}=I_{6}$ , meaning any secondary wrench is allowed. If the secondary manipulator is pushing against a flat surface, then $C_{2}$ may only contain the vector normal to the surface, and the optimisation would include an additional inequality constraint to allow only pushing in one direction, given by

$$C_{2}h_{e_{2}}\geq 0.$$ (46)

The above bi-level optimisation problem again separates the wrench maximisation problem into a linear lower-level optimisation and a low-dimensional higher-level problem. Again, the lower-level problem is solved using the dual-simplex method in MATLAB’s linprog function, and the upper-level problem uses simulated annealing, followed by the interior-point method to effectively search the highly non-linear space for an approximate global optimum.

### 3.7 Regrasping

**FIGURE 5:**
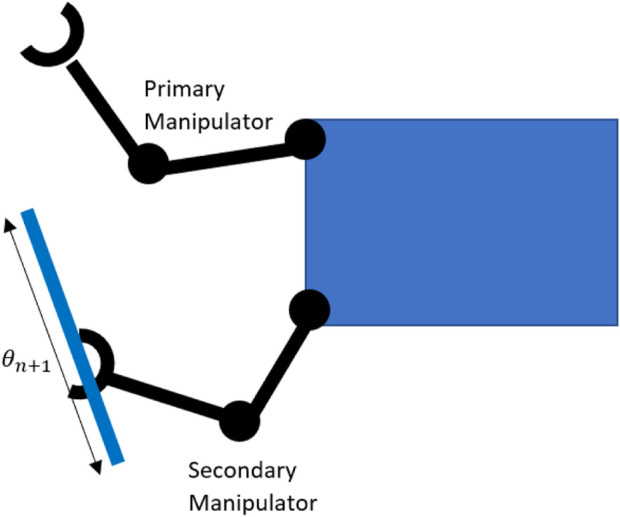
Parametrised regrasp points for second manipulator adding an additional kinematic variable $\theta_{n+1}$ .

The above case considered grasping a fixed point with a second arm. Generally, multiple grasping points will be available. We consider cases where these regrasp points can be parameterised to provide extra dimensions over which to optimise. Figure 5 shows a simple 2D example, where a one-dimensional set of parameterised re-grasping points result in an additional kinematic DOF, which can be optimised over. This leads to an additional DOF in the parameterised redundancy space $\theta_{r}$ in the above optimisation problem. Section 6 further describes the possible parametrisation of re-grasping points.

## 4 Wrench maximisation over a trajectory

In this section, a predefined end effector trajectory is considered, with corresponding desired wrench directions which should be maximised along the path. Only a UVMS with a single manipulator is considered, yet the analysis can be easily extended to multiple manipulators. Use cases for this method may be during a valve-turning operation, which requires maximising torque along the same direction throughout, or a waterjet blasting operation around a pipe, which requires resisting large forces normal to the pipe surface along the path. Dynamical effects due to velocities and accelerations cannot be ignored in this case. It is assumed that there is a given fully defined end effector pose trajectory parameterised in time and that the velocities at the start and end are zero. To maximise the capability of the system along this path, the objective is to maximise the minimum wrench capability along this trajectory.

### 4.1 Problem formulation

In order to make the problem tractable, the trajectory of the end effector is discretised into a set of k successive poses $x_{1},\ldots x_{k}$ , written as the stacked vector x , with corresponding redundant configurations $\theta_{r,1},\ldots \theta_{r,k}$ written as $\theta_{r}$ , giving corresponding system configurations $\theta_{1},\ldots \theta_{k}$ , written as θ . Each point along the trajectory of total time T is equally separated by Δt=T/k . At each end effector pose is a corresponding end effector velocity ${\dot{x}}_{1},\ldots {\dot{x}}_{k}$ written as $\dot{x}$ , which is computed using the timestep and differences between successive poses in SE(3) . Dynamic quantities are computed using the finite difference operator

$$D_{i}=\frac{1}{\Delta t}\left(\begin{array}{ccccc} -1 & 1 & & & \\ & -1 & 1 & \\ & & \ddots & \ddots & \\ & & & -1 & 1\\ & & & & 0 \end{array}\right)⊗I_{i\times i},$$ (47)

where ⊗ represents the Kronecker product and $I_{i\times i}$ is the i×i identity matrix. For this work, Equation 16 is slightly modified to give Equation 48

$$\dot{\theta }=J_{E}\dot{x}+Z_{r}\dot{\theta_{r}},$$ (48)

with Equation 49

$$J_{E}=\left(\begin{array}{c} J_{x}^{-1}\\ 0_{\left(n-6\right)\times 6} \end{array}\right),$$ (49)

where $J_{x}^{-1}$ is the inverse of $J_{x}\in R^{6\times 6}$ , which is the Jacobian for all DOFs which do not correspond to those is the parameterised redundancy space. As before, $Z_{r}\in R^{n\times \left(n-6\right)}$ is the null space projection matrix, which maps redundant velocities to system velocities, according to parameterisation. This formulation allows velocities for tracking end effector trajectories to be completely separated from redundant space velocities and *vice versa*. Now, we can rewrite Equation 48 as Equations 50, 51

$$\dot{\theta }=J_{E}\dot{x}+Z_{r}\dot{\theta_{r}}=J_{E}\dot{x}+Z_{r}D_{n-m}\theta_{r},$$ (50)

$$\ddot{\theta }=D_{n}\dot{\theta },$$ (51)

where $J_{E}$ and $Z_{r}$ are the stacked matrices formed from $J_{E}$ and $Z_{r}$ , respectively, at each configuration. Here, $D_{n-m}$ and $D_{n}$ are the finite difference operators acting on vectors of size (n−m) and n respectively, as defined in Equation 47.

At each timestep, the wrench is given by $h_{e,1},\ldots h_{e,k}$ , written as $h_{e}$ , and actuator efforts $u_{1},\ldots u_{k}$ , written as u . Each timestep also has a unit vector ${\hat{c}}_{1},\ldots {\hat{c}}_{k}$ along which the wrench should be maximised, written as $\hat{c}$ . The aim is to solve for a set of redundant configurations along the trajectory which maximises the minimum wrench capability, a max–min optimisation. Again, the problem can be greatly simplified by separation into a bi-level optimisation problem and through the use of redundancy parametrisation.

The trajectory optimisation problem can be written as the following bi-level optimisation problem

$${\theta_{r}}_{\mathrm{opt}}=\arg\underset{\theta_{r}}{\min}-f\left(\theta_{r}\right),$$ (52)

where ${\theta_{r}}_{\mathrm{opt}}$ is the optimal set of redundant configurations along the trajectory and $f\left(\theta_{r}\right)$ is the max–min wrench capability. There are constraints on the configurations in Equation 53

$${\theta_{r}}_{\min}\leq \theta_{r}\leq {\theta_{r}}_{\max},$$ (53)

where ${\theta_{r}}_{\min}$ and ${\theta_{r}}_{\max}$ are the minimum and maximum limits on the configurations along the trajectory, respectively, and constraints on the velocities in Equation 54

$${\dot{\theta_{r}}}_{\min}\leq \dot{\theta_{r}}\leq {\dot{\theta_{r}}}_{\max},$$ (54)

where ${\dot{\theta_{r}}}_{\min}$ and ${\dot{\theta_{r}}}_{\max}$ are the minimum and maximum limits on the velocities, respectively. The collision constraint in Equation 20 also applies at each timestep. Given a set of redundant configurations $\theta_{r}$ , the max–min wrench capability can be solved as a linear program (LP) over the entire trajectory, giving the lower level of the above bi-level optimisation in Equation 55

$$f\left(\theta_{r}\right)=\underset{p,u,h_{e}}{\max}\;p$$ (55)

subject to Equation 56

$$p<{\hat{c}}^{T}W_{h}h_{e},$$ (56)

where p represents the minimum wrench capability over the trajectory and $W_{h}$ is the wrench scaling metric, as before, for each timestep. The equality constraints are given by the dynamics balance equations at each timestep i , given by

$$\tau_{d,i}+J_{i}^{T}h_{e,i}=Bu_{i},$$ (57)

where $\tau_{d,i}$ and $J_{i}$ are the vector of dynamics terms $\tau_{d}$ and the Jacobian J , respectively, at timestep i . Since the terms in $\tau_{d}$ are fully determined by $\theta ,\dot{\theta }$ and $\ddot{\theta }$ , the dynamics terms can be predetermined over the entire trajectory independently of $h_{e}$ and u . There are inequality constraints

$$u_{\min}\leq u\leq u_{\max},$$ (58)

where $u_{\min}$ and $u_{\max}$ are the stacked vectors of minimum and maximum actuator efforts, respectively. The orthogonal wrench constraint is again given by

$$\left(\hat{c}{\hat{c}}^{†}-I_{6k}\right)W_{h}h_{e}=0.$$ (59)

As before, this constraint can be relaxed under certain conditions. Finally, there is a constraint on large changes to actuator efforts throughout the trajectory given by

$$-\Delta u_{\max}\Delta t\leq D_{mk}u\leq \Delta u_{\max}\Delta t,$$ (60)

where $\Delta u_{\max}$ is the maximum allowed actuator change per second and $D_{mk}$ is the finite difference operator acting on the vector of size (mk) . This accounts for the relatively slow dynamics of underwater thrusters. Again, the LP is solved using the dual-simplex method in MATLAB’s linprog function.

This is again a bi-level optimisation problem, with the linear program in Equation 55 as the lower-level optimisation. Changes to $\theta_{r}$ effect only the dynamics terms $\tau_{d},\dot{\theta },\ddot{\theta }$ and the Jacobian $J_{i}$ in Equation 57. Therefore, only the equality constraints are changed with changes in $\theta_{r}$ . Using the Karush–Kuhn–Tucker (KKT) conditions, the gradient of $f\left(\theta_{r}\right)$ with respect to $\theta_{r}$ can be found as Equation 61

$$\frac{df\left(\theta_{r}\right)}{d\theta_{r}}=\lambda_{eq}^{T}{\left.\frac{dc_{eq}}{d\theta_{r}}\right|}_{\begin{array}{c} \left(u^{*},h_{e}^{*}\right) \end{array}},$$ (61)

where $\lambda_{eq}$ is the vector of Lagrange multipliers associated with the equality constraints, and the final term on the right is the gradient of the equality constraints $c_{eq}$ , evaluated with the arguments of the solution to the lower-level LP problem $\left(u^{*},h_{e}^{*}\right)$ . This is a non-convex objective with multiple local minima. An interior-point solver using the MATLAB function fmincon is used and initiated at several different starting points in an attempt to find a good global minimum. For the system considered in the experiments in this work, this problem is solvable in several seconds on standard laptop hardware, making it amenable to real-time implementation (assuming the desired end effector trajectory and wrench maximization direction are defined).

Given a dynamically infeasible trajectory due to large dynamics terms, the lower-level LP cannot find a feasible solution, which satisfies the constraints in Equation 57. In this case, the lower-level problem is set to return a max–min wrench of $f\left(\theta_{r}\right)=\frac{1}{2}\tau_{d}^{T}\tau_{d}$ , and gradient $\tau_{d}$ to the upper-level problem, pushing the optimiser toward dynamically feasible trajectories.

### 4.2 Tracking dynamic trajectories

The above method finds a trajectory with smoothly varying actuator efforts, which maximises the minimum wrench in a given desired direction along the entire trajectory. The actual reaction forces and torques between the end effector and the environment during this trajectory will not be the same as those in the LP solution. Since the actuator constraints form a convex set, any value for $c^{T}h_{e}$ less than the maximum found by the optimisation will still fall within the feasible set. Additionally, assuming smooth changes in the interaction wrenches with the environment, and given the linear mapping between wrenches and each DOF, the smoothness properties are also conserved. The problem is finding the actuator efforts at a given timestep to track the desired trajectory, given that the actual efforts u may not match the LP solution. At a given timestep i , the control effort $\tau_{c}$ to track the trajectory is computed using an appropriate controller (Marais et al., 2021). Given the overactuated system, the objective is to find the actuator efforts $u_{c}$ which are close to the optimal LP solution $u_{i}^{*}$ , posed as a quadratic cost in Equation 62

$$\underset{u_{c}}{\min}{\left(u_{c}-u_{i}^{*}\right)}^{T}Q\left(u_{c}-u_{i}^{*}\right),$$ (62)

where Q is simply a diagonal weighting matrix, with the equality constraint Equation 63

$$Bu_{c}=\tau_{c},$$ (63)

which is simply the actuator model. Using the method of Lagrange multipliers, this has solution in Equation 64

$$u_{c}^{*}=u_{i}+B_{Q}^{†}\left(\tau_{c}-Bu_{i}\right),$$ (64)

where $B_{Q}^{†}$ is the weighted pseudoinverse given by Equation 65

$$B_{Q}^{†}=Q^{-1}B^{T}{\left(BQ^{-1}B^{T}\right)}^{-1}.$$ (65)

This result does not incorporate the inequality constraints on the actuators. These are in Equation 66

$$u_{\min}\leq u_{i}\leq u_{\max},$$ (66)

which account for actuator effort limits and Equation 67

$$-\Delta u_{\max}\Delta t\leq D_{m}u_{i}\leq \Delta u_{\max}\Delta t,$$ (67)

which account for actuator effort rate change limits. This is a quadratic programming problem, which can be solved efficiently in real time for control.

## 5 Maximum wrench impulse

This section looks at generating a maximum momentary wrench for a fixed end effector pose, using dynamic vehicle manipulator motions while keeping the end effector fixed. A use case for this method may be for shifting a very heavy weight or a stuck valve. Humans naturally perform these motions for similar tasks, where the momentum of the person is used to momentarily generate large forces.

### 5.1 Problem formulation

For a given fixed end effector pose and set of redundant configurations which define a dynamic trajectory, the maximum wrench problem is given by Equation 68

$$f=\underset{u,h_{e}}{\max}\;\max\left(c^{T}W_{h}h_{e}\right),$$ (68)

which is a linear max−max problem. These problems can be reformulated as a mixed-integer linear program (MILP) using the big-M method, although this is no longer a convex optimisation problem. Since this is used as the lower-level problem in a bi-level optimisation framework, efficient solutions are required. To resolve this, the maximum wrench impulse can be chosen to occur at an arbitrarily chosen specific timestep $t_{h}$ , giving Equation 69

$$f=\underset{u,h_{e}}{\max}\;\left(c_{t_{h}}^{T}W_{h}h_{e,t_{h}}\right).$$ (69)

All constraints from the dynamic wrench maximisation in Equations 57–60 also apply. This is a linear program which again is the lower-level problem for the bi-level trajectory optimisation with upper level given by Equation 52, with the solution and gradients found in the same way as before.

## 6 Results

We present the wrench maximisation results for the 4DOF manipulator and 6DOF vehicle shown in Figure 1. This system has a degree of redundancy of 4. Two wrench objectives are compared, torque along the *z* direction simulating turning a valve, and force in the *z* direction, which tests lifting a heavy load. The torque limit on each joint is [±9,±9,±9,±2]Nm , and each thruster has an asymmetrical thrust limit of −40 N–50 N. The force of each thruster can vary by 50 N/s, while the rate of change of joint torque is effectively instantaneous. Six load cells were attached in series, as shown in Figure 1, allowing all six components of the applied wrench to be measured simultaneously. The sensor setup was calibrated using known masses and levers. Due to the consistency of the results, only the results of a single run for each experiment are shown, although a minimum of two runs were completed for each case to confirm consistency.

### 6.1 Static wrench capability

**FIGURE 6:**
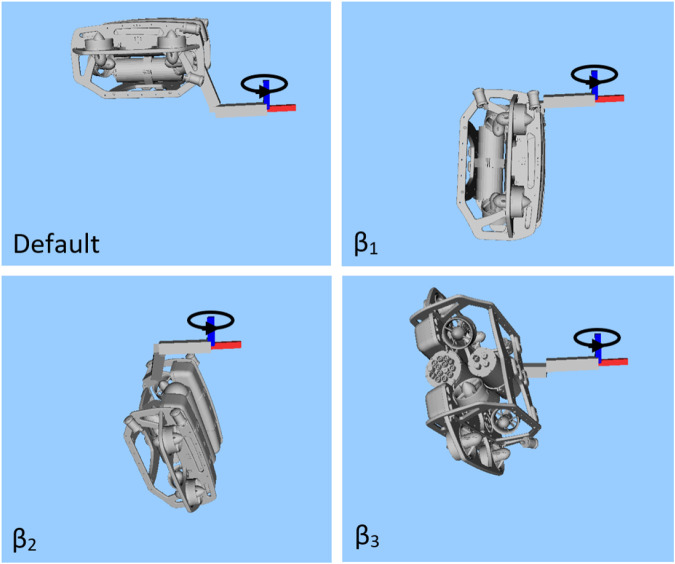
Optimal static configuration for torque in the *z*-direction (blue axis), comparing the default upright case, to the solutions which optimise $\beta_{1}$ , $\beta_{2}$ , and $\beta_{3}$ .

For the case of static torque maximisation in the *z* direction, four configurations are compared, shown in Figure 6. First is the default configuration, which is simply the manipulator in a neutral position with the vehicle upright. The three other cases are the optimised configurations for $\beta_{1},\beta_{2}$ and $\beta_{3}$ solved using the bi-level optimisation approach, as described in Section 3, with the optimal thruster forces and manipulator torques computed accordingly.

**FIGURE 7:**
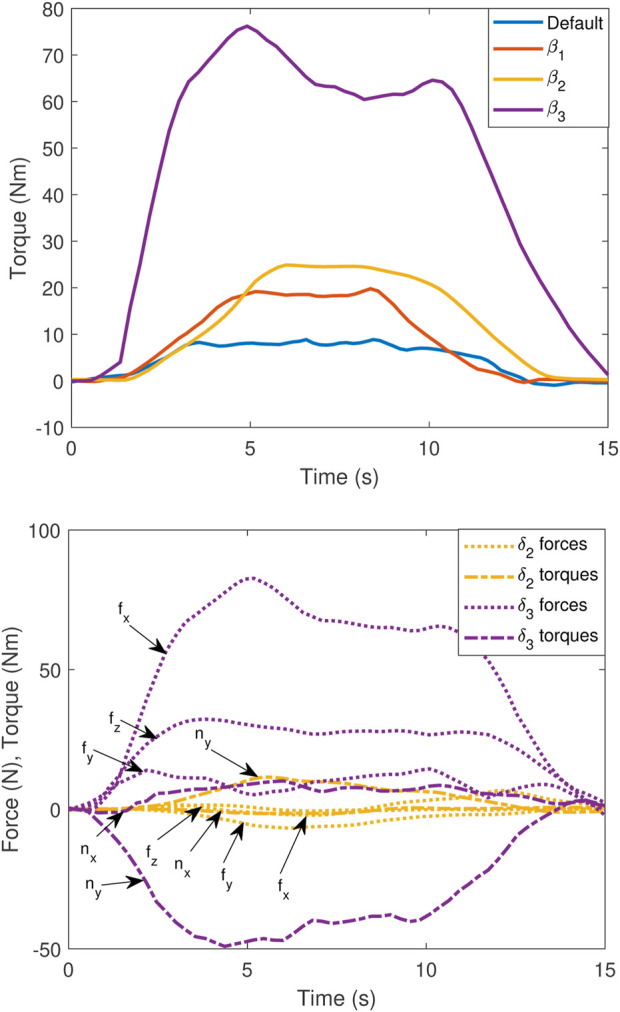
(Top) Torque along the *z* direction during static wrench maximisation, comparing the default, $\beta_{1}$ , $\beta_{2}$ , and $\beta_{3}$ . (Bottom) Forces f in xyz and torques n in xy during static torque maximisation in the *z* direction, comparing $\beta_{2}$ and $\beta_{3}$ .

Figure 7 (top) shows the torque along the *z* direction, as measured by the sensor setup during the experiments for each of the four configurations. Each experiment consists of a 5-s ramp-up and ramp-down and a 5-s maximum wrench.

The default case is limited to a torque of 9 Nm since the *z*-axis is aligned with the base joint of the manipulator in this case. The standard approach of optimising for the transmission ratio $\beta_{1}$ (Faroni et al., 2016; Nemec, 1997) results in a maximum torque of approximately 19.5 Nm, while the proposed bi-level optimisation approach for $\beta_{2}$ yields a maximum of 25 Nm. Finally, removing the constraint orthogonal wrench components for $\beta_{3}$ leads to a maximum torque of 76 Nm. The inconsistent torque readings during the fixed maximum thrust period are due to the effects of swirling water after several seconds in the relatively small test tank.

The default, $\beta_{1}$ and $\beta_{2}$ , cases all have constraints on wrenches orthogonal to torque along the *z* direction. The $\beta_{3}$ case relaxes this constraint and has significant orthogonal components. Figure 7 (bottom) shows each of the three orthogonal forces and two orthogonal torques during the experiment. All the orthogonal components during the $\beta_{2}$ experiment are relatively small, with a peak of approximately 10 Nm along the *y* direction due to flexible strain in the sensor and UVMS setups. In contrast, the $\beta_{3}$ experiment has significant forces of approximately 80 N and torque of approximately 50 Nm. These may be acceptable in a case such as turning a valve, where the valve can resist these orthogonal wrenches.

**FIGURE 8:**
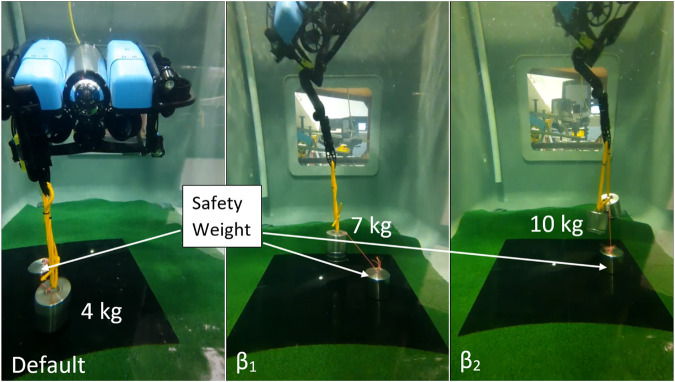
Experiments comparing the default, $\beta_{1}$ and $\beta_{2}$ force maximisation cases, showing the maximum lifted test mass. Each test mass is attached to a safety weight through a slack purple line to keep the system constrained after the test mass is lifted.

For the case of static force maximisation in the *z* direction, three configurations are compared, shown in Figure 8. Again, the first is the default configuration, followed by optimal configurations for $\beta_{1}$ and $\beta_{2}$ . In this case, optimising using $\beta_{3}$ is not valid as the weight should be lifted vertically up and not accelerated in orthogonal directions. This test was performed using known weights, and the successfully lifted masses are shown in Figure 8. The default case can lift a maximum of 4 kg, the transmission ratio method $\beta_{1}$ can lift 7 kg, and the proposed method $\beta_{2}$ can lift 10 kg.

The results for both the torque maximisation and force maximisation experiments show significant increases of approximately 30% and 40%, respectively, in wrench capability when comparing optimisation using the transmission ratio $\beta_{1}$ , as compared to the proposed method of optimising the directional wrench polytope using a bi-level optimisation with a LP lower-level problem $\beta_{2}$ . Additionally, in the case when the orthogonal wrench constraints can be removed, the experimental torque capability increased threefold.

### 6.2 Multiple contact points

**FIGURE 9:**
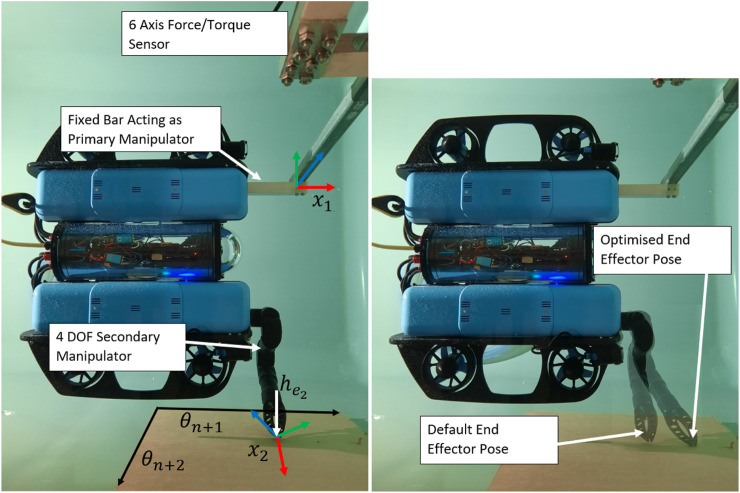
(Left) Experimental setup for multiple contacts points. A fixed bar is used as the primary manipulator, with the end effector pose $x_{1}$ , and a 4DOF manipulator is used as the secondary manipulator with end effector pose $x_{2}$ . The secondary manipulator is contacting the side of the tank where a normal force into the wall can be applied, labelled as $h_{e_{2}}$ . The range of possible regrasping points for the second manipulator, which provide additional DOFs over which to optimise are labelled as axes $\theta_{n+1}$ and $\theta_{n+2}$ . (Right) Default and optimised configurations and end effector poses for a secondary contact point during torque maximisation.

The experimental setup for multiple contact point is shown in Figure 9 (left). A fixed bar is used as the primary manipulator with end effector $x_{1}$ and is attached to the 6-axis force–torque sensor setup. A 4DOF manipulator is used as the secondary manipulator with the end effector pose $x_{2}$ , which is shown in contact with the side of the tank where a normal force can be applied. The first constraint in Equation 45 is also applied, limiting the wrench applied at $x_{1}$ to a pure torque in the *z* direction. It is assumed all forces and torques at $x_{2}$ are 0 except for a positive normal force into the wall. Therefore, $C_{2}$ only has one component and the second constraint in 45 applies, as well as in inequality constraints in Equation 46, to ensure the contact force is into the wall.

**FIGURE 10:**
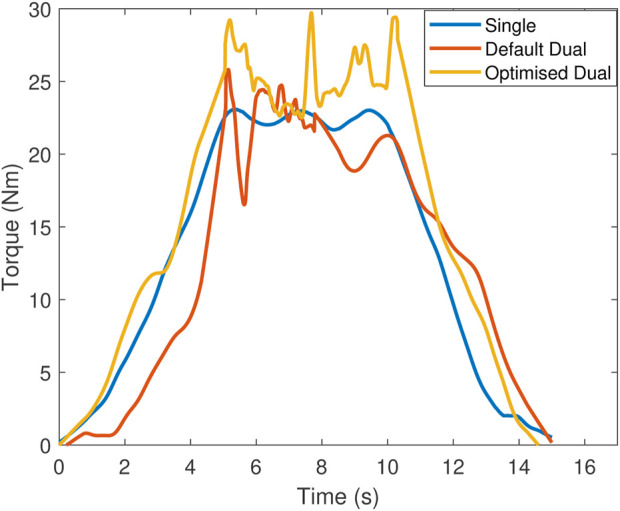
Torque along the *z* direction during static wrench maximisation comparing single manipulator, dual manipulators in the default end effector pose, and dual manipulators in the optimised end effector pose.

Figure 9 shows the set of possible regrasping points which provide two additional DOFs over which to optimise. Figure 10 shows the results for torque maximisation along the *z* direction, comparing the single and dual manipulator cases. For the dual manipulator case, both the default end effector pose and the optimised end effector pose are tested, shown in Figure 9 (right). The single manipulator torque reaches a maximum of 23 Nm, the default dual case reaches 26 Nm, and the optimised dual case reaches 30 Nm, an increase of 13% and 30%, respectively. The data for this experiment contain more noise since the vehicle is very close to the edge of the tank by necessity, causing large effects from swirling water in the tank. These effects may be significant near solid structures in the underwater environment, requiring additional modelling which is left as future work.

### 6.3 Wrench maximisation over a trajectory

**FIGURE 11:**
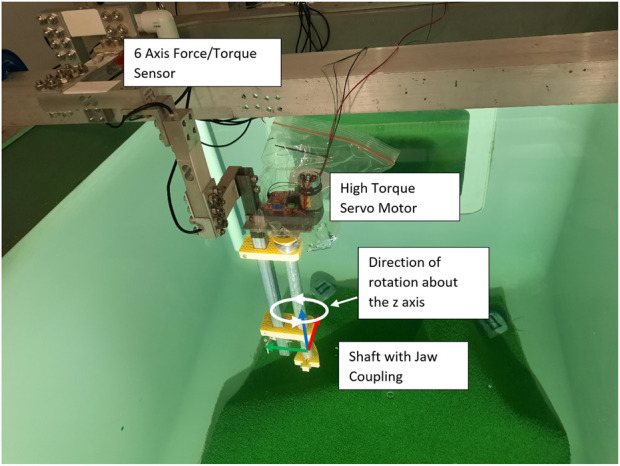
Setup for testing dynamics wrenches over a trajectory, using a servo motor and attached shaft to simulate a turning valve.

The experimental setup for testing wrenches over a rotating end effector trajectory along the global *z*-axis is shown in Figure 11. This simulates turning a valve along this direction of rotation. A high-torque servo motor above the water line is used to generate the controlled rotation and is attached to a shaft, which reaches into the tank. By controlling the rotation of the simulated valve, the maximum wrench capability throughout the trajectory can be accurately determined. The end effector of the UVMS attaches to a jaw coupling on the end of the shaft, and the whole setup is mounted to the end of the 6-axis force–torque sensor.

**FIGURE 12:**
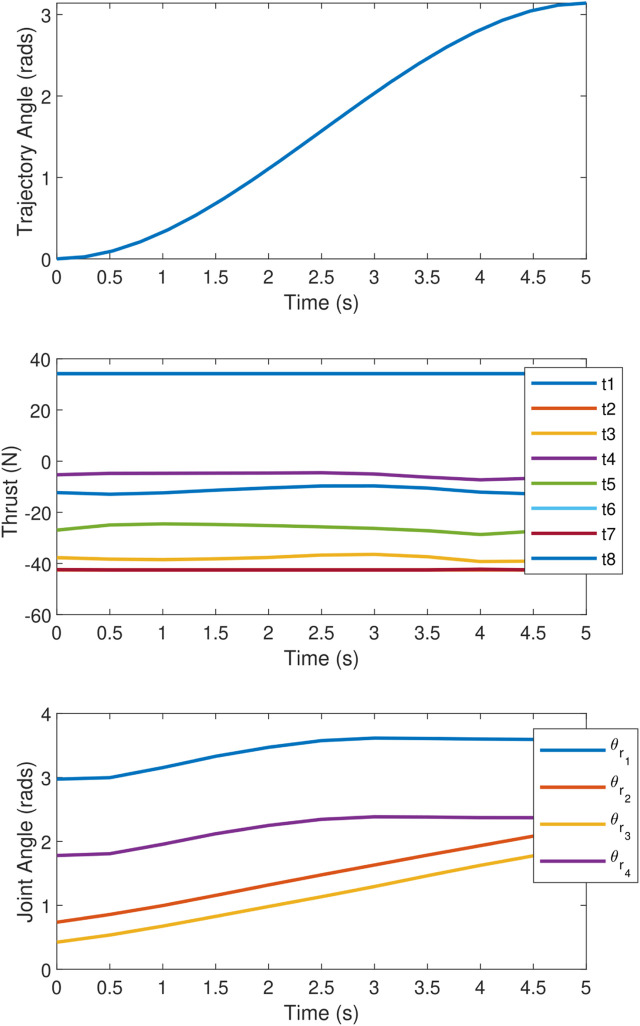
Plots during the rotating end effector trajectory showing (top) the end effector angle, (middle) vehicle thrust forces, and (bottom) redundant joint angles.

The objective which is tested is maximising the torque along the *z*-axis with no orthogonal wrench components, while the end effector tracks a 180° rotating trajectory. The trajectory is shown in the top plot of Figure 12 and is generated as a cubic spline with a velocity of 0 at each end point, with a total time of 5 s. Ten points along the trajectory are considered; therefore, k=10 for the trajectory optimisation problem described in Section 4. The values for each joint angle and thruster force were interpolated linearly between each timestep when sending commands to the UVMS during the experiment. The middle plot in Figure 12 shows the thruster forces throughout the trajectory, showing the effect of the constraint in Equation 60, which limits large changes in actuator effort. Finally, the bottom plot in Figure 12 shows the joint angles which correspond to the redundant DOF, showing a relatively smooth change in redundant configuration throughout the rotation.

**FIGURE 13:**
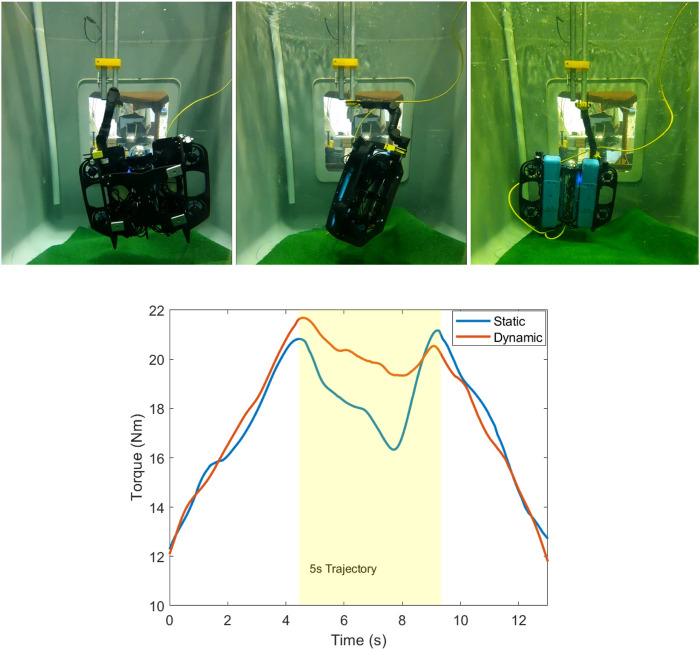
(Top) Sequence of images during the 5-s 180° rotating trajectory, showing the changing manipulator joint angles during the rotation. The images are at 0° , 90° , and 180° . (Bottom) Torque along the *z* direction during a dynamic rotating end effector trajectory, a comparison of the static and dynamic redundant configuration cases.

Figure 13 (top) shows a sequence of images of the UVMS during the experiment at 0° , 90°, and 180° , showing the changing joint angles throughout.

Figure 13 (bottom) shows the results of the torque along the *z*-axis, comparing the dynamic case which tracks the optimised trajectory, and the static case which maintains a fixed redundant configuration throughout. The static redundant configuration which is chosen is the configuration which maximises the static torque capability using $\beta_{2}$ . The static case achieves a minimum torque of 16 Nm throughout the 5-s rotation period, while the dynamic trajectory achieves a minimum torque of 19 Nm, a 19% increase. The results show that consideration of the dynamical effects during the rotating end effector trajectory leads to a significant increase in the max–min torque.

Since this rotation is about the vertical axis, the effects of changing gravity and buoyancy vectors in the UVMS frame due to the end effector rotation are not present. Therefore, the improved performance of the dynamic case, as compared to the static case, is purely due to consideration of dynamical effects from velocity and acceleration. The difference in performance between the dynamic trajectory optimised case and the static case is likely to be much more significant with end effector trajectories which include rotations about a non-vertical axis.

### 6.4 Maximum wrench impulse

**FIGURE 14:**
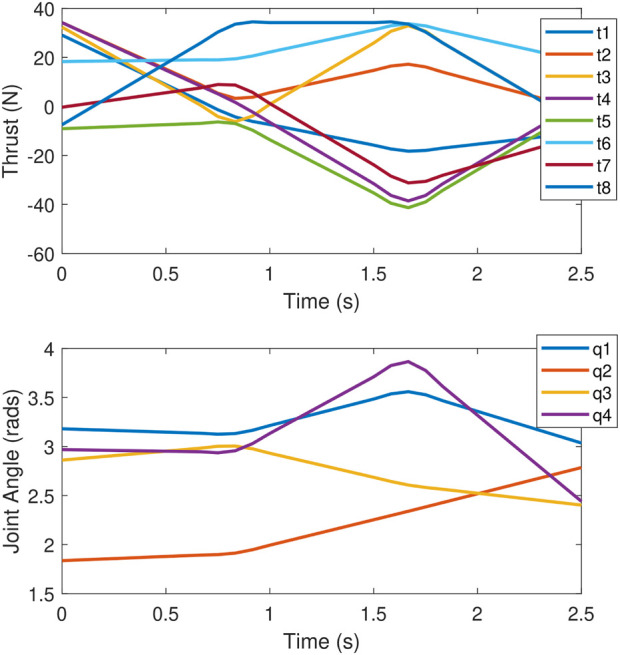
Plots during the maximum wrench impulse trajectory showing (top) the vehicle thrust forces and (bottom) redundant joint angles.

**FIGURE 15:**
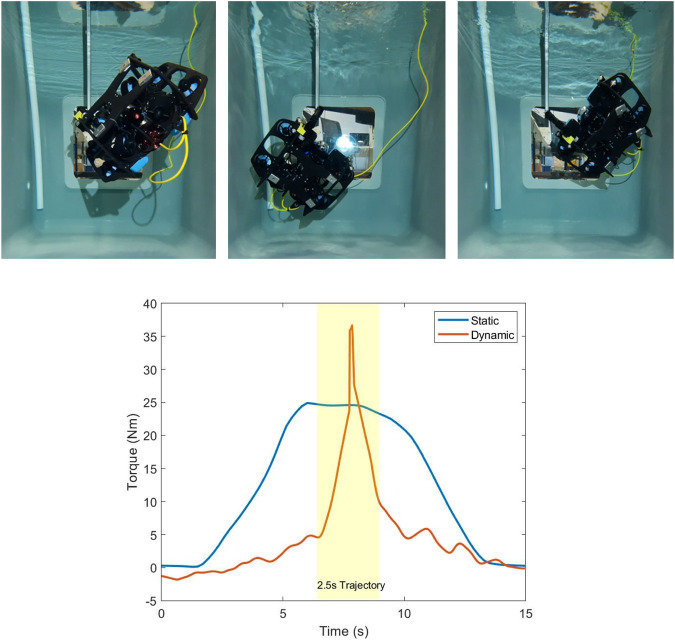
(Top) Sequence of images during the 2.5-s maximum impulse trajectory. Resultant trajectory is highly dynamic, allowing a large wrench impulse to be generated. (Bottom) Torque along the *z* direction during a heaving motion, compared to the static $\beta_{2}$ case.

The experimental setup for maximum wrench impulse trajectories is the same as the static wrench maximisation setup shown in Figure 1. Again, the objective is to maximise the torque along the *z* direction with no orthogonal wrench components, in this case by using dynamic motions to generate a large torque impulse momentarily, while keeping the end effector fixed. A trajectory of 2.5 s with four intermediate configurations was chosen; therefore, k=4 for the trajectory optimisation problem is described in Section 5. The third timestep is chosen as the point of maximum torque; therefore, $t_{h}=3$ . As before, values for each joint angle and thruster force are interpolated linearly between each timestep when sending commands to the UVMS during the experiment. Figure 14 shows the results of the trajectory optimisation, with the top plot showing the thruster forces throughout the trajectory and the bottom plot showing the joint angles. The resulting trajectory leads to both joint velocities and thruster rates of change at the limits set in the optimisation problem, leading to a highly dynamic trajectory. Figure 15 (top) shows a sequence of images during the trajectory, and Figure 15 (bottom) shows a plot of the measured torque results. The plot compares the static case, which is identical to the $\beta_{2}$ optimised results from Figure 7, compared to the dynamic impulse case. The dynamic trajectory generated a momentary torque impulse of 35 Nm, which is a 40% torque improvement over the best static results with orthogonal wrench constraints.

## 7 Conclusion

This work focuses on maximising the maximum wrench capability of UVMS. A bi-level optimisation method is proposed for maximising static wrenches, and experimental results show a significant improvement over optimising the transmission ratio. Further results show that relaxing constraints on orthogonal wrenches lead to significant increases in the wrench capability in relevant use cases. The case of multiple contact points, as well as re-grasping of secondary points, is also considered, with experimental results again showing an increased wrench capability. A similar bi-level optimisation approach is introduced for max–min wrench optimisation over a trajectory, with experimental results confirming the validity of the method. Finally, a method is proposed for finding dynamic trajectories which generate large wrench impulses, with supporting experimental results. Further work is required for dealing with the effects of self-generated currents by the vehicle thrusters when operating near underwater structures. Additional work would look at automatic recognition of viable secondary contact points. Further experimental results are required to test the trajectory optimisation method for end effector trajectories which contain rotation about a non-vertical axis. Finally, further work is required to implement the proposed methods in a truly real-time implementation (rather than just pre-solving the optimisation several seconds before). This would allow for online configuration adjustments during application of an end effector wrench, for example, to reduce end effector tracking errors.

## Data Availability

The raw data supporting the conclusions of this article will be made available by the authors, without undue reservation.
